# Parenteral Ozone Therapy Combined with Topical Antimicrobials Enhances Tissue Repair in Mandibular Osteonecrosis of Elderly Female Rats

**DOI:** 10.3390/jfb17080378

**Published:** 2026-08-03

**Authors:** Izabela Fornazari Delamura, Melissa Koto Murai, Arthur Henrique Alécio Viotto, Ana Maira Pereira Baggio, Natália Saori Izumi, Douglas Sadrac de Biagi Ferreira, Vinicius Ferreira Bizelli, Emanuele di Edoardo, Ignazio Scuto, Pietro Montemezzi, Leonardo Perez Faverani, Carlos Fernando Mourão, Ana Paula Farnezi Bassi

**Affiliations:** 1 Department of Diagnosis and Surgery, Aracatuba School of Dentistry, Sao Paulo State University (UNESP), Aracatuba 16015-050, SP, Brazil; 2 Department of Biosciences, Piracicaba Dental School, University of Campinas (UNICAMP), Piracicaba 13414-903, SP, Brazil; 3 IRCCS San Raffaele Hospital, Via Olgettina 60, 20132 Milan, Italy; 4 Department of Oral Diagnosis, Division of Oral and Maxillofacial Surgery, Piracicaba Dental School, University of Campinas (UNICAMP), Piracicaba 13414-903, SP, Brazil; 5 Department of Basic and Clinical Translational Sciences, Tufts University School of Dental Medicine, Boston, MA 02111, USA

**Keywords:** osteoporosis, ozone, osteonecrosis associated with bisphosphonates, rats

## Abstract

Medication-related osteonecrosis of the jaws (MRONJ) remains a challenging complication associated with antiresorptive therapy, highlighting the need for effective adjunctive preventive strategies. This study evaluated the effects of systemic ozone therapy, alone or combined with topical 10% metronidazole paste or 2% chlorhexidine gel, on alveolar repair in a zoledronate-induced rat model of MRONJ. Forty-two ovariectomized female Wistar rats were allocated into seven experimental groups. Following mandibular first molar extraction, animals received systemic ozone therapy and/or topical antimicrobial treatment according to the experimental protocol. Clinical, histomorphometric, micro-computed tomography (micro-CT), Picrosirius Red, and immunohistochemical analyses were performed. Zoledronate treatment markedly impaired alveolar repair, resulting in reduced new bone formation, increased non-vital bone, epithelial disorganization, and unfavorable bone microarchitecture. Systemic ozone therapy significantly increased new bone formation and improved bone microstructural parameters compared with untreated zoledronate animals. Ozone-treated groups also exhibited more organized collagen fibers and increased VEGF and osteocalcin immunostaining. Topical metronidazole and chlorhexidine were associated with favorable tissue repair findings, while combined therapies showed more favorable outcomes than zoledronate treatment alone. These findings suggest that systemic ozone therapy, particularly when associated with topical antimicrobial treatments, improves alveolar repair and represents a promising adjunctive strategy for preventing MRONJ.

## 1. Introduction

Bisphosphonates (BPs) and anti-RANKL monoclonal antibodies are antiresorptive drugs that inhibit osteoclastic activity and act on bone remodeling [1,2]. They are highly effective in the treatment of several bone diseases, such as osteoporosis, Paget’s disease, osteogenesis imperfecta, multiple myeloma, bone pain management, the regulation of hypercalcemia, and the inhibition of the progression of metastasis in osteotropic malignancies [3]. Treatment plans involving the use of BPs including dosage, duration, frequency, and type of BP vary depending on the specific pathology being treated [3,4].

The American Association of Oral and Maxillofacial Surgeons (AAOMS) and position papers worldwide define medication-related osteonecrosis of the jaws (MRONJ) as exposed bone in the maxillofacial region persisting for more than 8 weeks [4,5,6,7,8]. MRONJ is diagnosed in patients undergoing antiresorptive therapy, alone or combined with immunomodulatory or antiangiogenic agents, without prior head and neck radiotherapy. Diagnosis is primarily clinical and often includes symptoms such as pain, swelling, foul odor, purulent discharge, and fistula formation [5,6,9,10]. Treatment options vary by disease stage and include 0.12% chlorhexidine gluconate rinses, prolonged antibiotics, laser therapy, hyperbaric oxygen, surgical debridement, and, in severe cases, partial mandibulectomy or maxillectomy. However, therapies aimed at biomodulation of osteonecrotic tissues lack consensus regarding indications, highlighting the need for further research [11,12,13].

Ozone (O_3_) may serve as an adjuvant therapeutic option and appears to aid in the treatment of these conditions. The role of O_3_ generated from air has been evaluated in some preclinical studies for the treatment of MRONJ based on the hypothesis that ozone could induce tissue repair by cleansing osteonecrotic lesions, leading to mucosal healing [14,15,16]. In systemic applications, studies have shown that ozone enhances bone and soft tissue regeneration by improving microcirculation and modulating immune responses. It has been demonstrated that ozone increases the effectiveness of both surgical and pharmacological treatments for MRONJ when administered before and after dental procedures [16,17,18].

Regarding topical treatments for osteonecrosis-affected areas, no established guidelines exist defining protocols to reduce infection, prevent new necrotic lesions, and improve quality of life in patients with MRONJ, who often experience painful symptoms [19]. Alternative therapies such as metronidazole-based paste, a well-established treatment, may help modulate these conditions [19]. Poi et al. [20,21] evaluated a paste containing 10% metronidazole and 2% lidocaine, formulated with lanolin or carboxymethylcellulose as a vehicle, for alveolitis management [20,21]. The literature findings suggest that metronidazole paste is a promising alternative, effectively alleviating pain, promoting bone neoformation, and providing the advantage of topical application, which achieves high local drug concentrations while minimizing systemic side effects.

Considering the therapeutic options available for patients affected by osteonecrosis regardless of disease stage, although there is no clear consensus, the literature consistently recommends the topical use of mouthwashes, particularly chlorhexidine, due to its bactericidal effect that helps control local contamination [21]. The use of 2% chlorhexidine gel has also been explored in cases of osteitis, demonstrating good effectiveness in bacterial control in contaminated areas [22,23].

Given the World Health Organization’s projection that the global population aged over 60 will exceed 2 billion by 2050, population aging is an undeniable consequence of recent demographic transitions. This trend necessitates that healthcare professionals deepen their understanding of age-associated diseases and explore alternative treatment strategies. Research on ozone therapy as an adjunct for bone repair, particularly in osteonecrosis, and its combination with topical therapies remains limited. Most studies lack consensus on treatment protocols, and few in vivo investigations exist that evaluate the biological effects of these therapies under conditions predisposing to medication-related osteonecrosis of the jaw (MRONJ).

## 2. Materials and Methods

### 2.1. Experimental Design

This preclinical study was conducted in accordance with the ethical principles for animal experimentation established by the National Council for the Control of Animal Experimentation (CONCEA, Brazil) and institutional guidelines for the care and use of laboratory animals. All experimental procedures complied with ethical standards to ensure animal welfare throughout the study. The study was designed, conducted, and reported in accordance with the ARRIVE 2.0 (Animal Research: Reporting of In Vivo Experiments) guidelines [24]. The protocol was reviewed and approved by the Animal Ethics Committee (CEUA) of the School of Dentistry of Aracatuba, São Paulo State University (UNESP), under protocol number FOA nº 0268-2022.

### 2.2. Sample Selection

Forty-two female Wistar rats (Rattus norvegicus) were obtained from the Central Animal Facility of the School of Dentistry of Araçatuba, São Paulo State University “Júlio de Mesquita Filho”—UNESP, at 12 months of age. The sample size was determined using the “Sample Size for ANOVA” tool in the SigmaPlot 12.0 software. Based on the study by Silva et al. (2022) [23] using a similar methodology, and considering the difference between two means (0.57) and a standard deviation value from a primary outcome analysis (histomorphometric analysis), a total sample of 42 animals was obtained. Considering the risk of animal loss, as supported by the National Council for the Control of Animal Experimentation (CONCEA), it was [25,26] decided to increase the sample size by one animal per group, resulting in *n* = 6 animals per group.

### 2.3. Experimental Surgery

The animals were housed in the Animal Facility of the Department of Diagnosis and Surgery at the School of Dentistry of Aracatuba under controlled conditions, in plastic cages (four per cage) with free access to water and standard feed. After an 8-day acclimatization period, all animals underwent bilateral ovariectomy. Anesthesia was induced with intraperitoneal injections of Ketamine (70 mg/kg; Francotar^®^, Virbac, Sao Paulo, Brazil) and Xylazine (10 mg/kg; Rompum^®^, Bayer, Sao Paulo, Brazil). Positioned in lateral recumbency, a 1.5 cm incision was made on each flank. Blunt dissection of subcutaneous tissue and peritoneum exposed the abdominal cavity. The ovaries and uterine horns were ligated with 4-0 Polyglactin 910 sutures (Vicryl™, Johnson & Johnson, New Brunswick, NJ, USA) and excised, and the incision was closed in layers using the same suture material [25,26].

The animals were randomly assigned into seven experimental groups (*n* = 6):

- **SAL group (SAL)** (Positive Control): Received 0.9% saline intraperitoneally (0.45 mL every 3 days for 7 weeks) [27].
- **ZOL group (ZOL)** (Negative Control): Received zoledronate 100 µg/kg intraperitoneally every 3 days for 7 weeks [28].
- **ZOL+OZ (OZ):** Same ZOL regimen plus systemic ozone (0.7 mg/kg, intraperitoneal) administered immediately, and on days 2 and 4 after tooth extraction [16,27].
- **ZOL+MTZ (MTZ):** Same ZOL regimen plus topical 10% metronidazole ointment applied on the day of extraction, and on days 2 and 4 postoperatively [28].
- **ZOL+MTZ+OZ (MTZ+OZ):** Same ZOL and MTZ regimens combined with systemic ozone (0.7 mg/kg) at the same postoperative intervals [16,27].
- **ZOL+CLX (CLX):** Same ZOL regimen plus topical 2% chlorhexidine gel applied on the day of extraction, and on days 2 and 4 postoperatively [28].
- **ZOL+CLX+OZ (CLX+OZ):** Same ZOL and CLX regimens combined with systemic ozone (0.7 mg/kg) at the same postoperative intervals [16,27].

The bilateral ovariectomy procedure and the postoperative interval adopted in this study followed a previously validated experimental protocol that has been consistently shown to induce estrogen deficiency and osteoporotic changes in rats [13,25].

**Figure 1:**
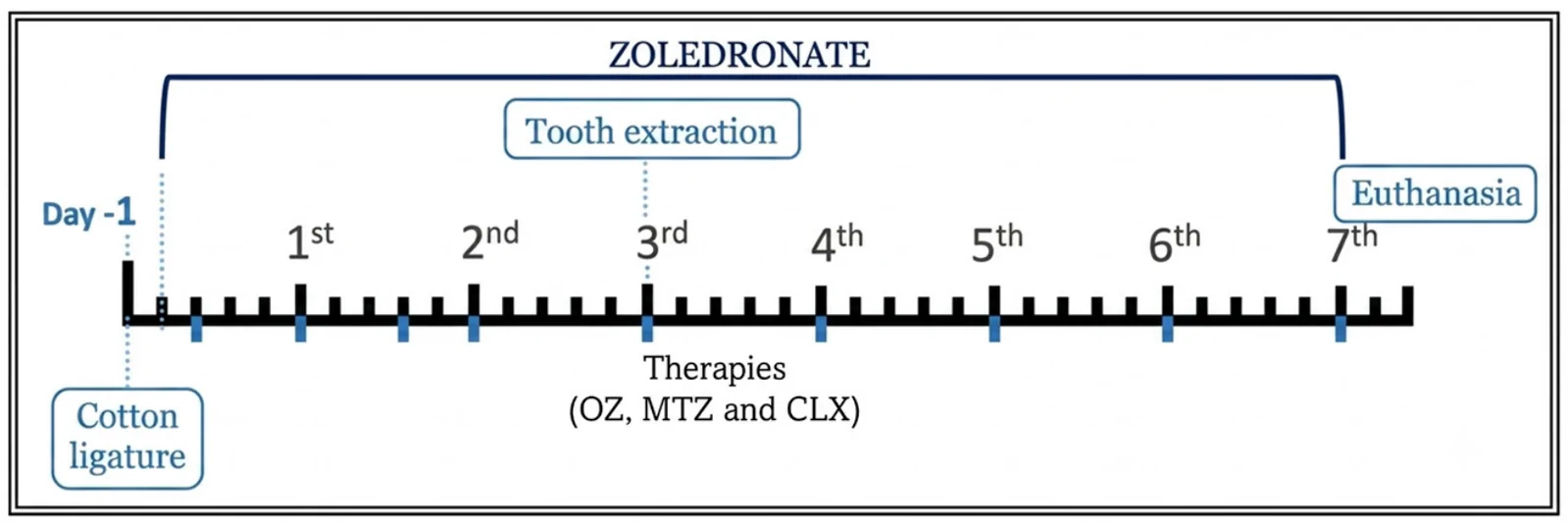
Schematic representation of the experimental design.

Sixty days after osteoporosis induction, the left mandibular first molar of all rats was ligated with cotton thread (day 1). Starting on day 1, osteonecrosis was induced via intraperitoneal injections of zoledronate (100 µg/kg in 0.45 mL saline) every 3 days for 7 weeks. In the third week, the ligature was removed, and the left mandibular first molar was extracted under sedation. After oral antisepsis with 10% povidone–iodine, syndesmotomy and tooth extraction were performed using instruments adapted for rats. All animals underwent the same procedure (Figure 1).

#### 2.3.1. Application of Local Therapies: Application of 10% Metronidazole Paste and 2% Chlorhexidine Gel

Following tooth extraction, the animals in the ZOL+MTZ, ZOL+OZ+MTZ, ZOL+CLX, and ZOL+OZ+CLX groups underwent topical application of 10% metronidazole paste and 2% chlorhexidine gel. The application was performed using a 5 mL syringe with a 27 G needle at three times: immediately after extraction and on the second and fourth postoperative days. The material was kept in place at the surgical site for two minutes. The animals continued receiving ZOL therapy until the time of euthanasia.

#### 2.3.2. Ozonotherapy

The rats in the ZOL+OZ, ZOL+OZ+MTZ, and ZOL+OZ+CLX groups received ozone therapy using a device supplied by OZONE&LIFE INDÚSTRIA, COMÉRCIO E SISTEMAS LTDA (São José dos Campos, SP, Brazil). Ozone was administered intraperitoneally at a dose of 0.7 mg/kg using a disposable 10 mL syringe fitted with a 25 mm × 0.70 mm needle. The intraperitoneal route was selected because it has been widely adopted in experimental studies investigating the systemic biological effects of ozone therapy in rats, providing a reproducible method for systemic administration and allowing the evaluation of its immunomodulatory and regenerative effects [29]. The selected dose (0.7 mg/kg) was based on recent dose–response studies demonstrating effective stimulation of bone repair and angiogenesis without evidence of systemic toxicity in experimental animal models [27,30,31]. Unlike topical administration, which primarily exerts local antimicrobial and wound-healing effects, systemic intraperitoneal ozone administration promotes the controlled generation of reactive oxygen species and lipid oxidation products, thereby inducing oxidative preconditioning, the activation of endogenous antioxidant systems, the modulation of inflammatory responses, improved microcirculation, and enhanced angiogenesis and tissue repair [17,32,33]. Additionally, they received topical application of metronidazole paste and 2% chlorhexidine gel. 

#### 2.3.3. Analyses

Euthanasia of the animals was performed by administering an overdose of anesthetic. The SAL, ZOL, ZOL+OZ, ZOL+CLX, ZOL+MTZ, ZOL+OZ+MTZ, and ZOL+OZ+CLX groups were euthanized 28 days after the tooth extraction procedure (totaling 7 weeks). Subsequently, the hemimandibles were carefully dissected and post-fixed for 24 h in 4% paraformaldehyde. Histological, histometric, inflammatory profile, immunohistochemistry, and statistical analyses were performed.

#### 2.3.4. General Health Condition Analysis and Extraoral and Intraoral Examination

All collected specimens were clinically evaluated, along with photographic records, to identify characteristics such as mucosal appearance, presence or absence of reparative tissue, coloration of soft tissues, presence or absence of exposed bone tissue, presence of bone sequestrum, suppuration, or any other aspects related to healing. In addition, the animals were weighed weekly to monitor their general health.

#### 2.3.5. Micro-CT Analysis

The samples were analyzed using high-resolution digital micro-computed tomography (micro-CT) with the SkyScan 1176 system (Bruker MicroCT, Aatselaar, Belgium, 2003). Scanning was performed using 8 µm slice thickness, at 90 kV and 111 µA, with a copper filter and a rotation step of 0.05 mm. The X-ray projections were stored and reconstructed using NRecon software (SkyScan, 2011; version 1.6.6.0), and the region of interest (ROI) was defined. Sample orientation and alignment were standardized with the Data Viewer software (SkyScan, version 1.4.4, 64-bit), enabling visualization in the transverse, longitudinal, and sagittal planes. The ROI was established as a 4 mm^3^ volume encompassing the area previously occupied by the mesial and distal roots of the left first lower molar, including surrounding tissues. Quantitative analysis was performed using CTAn software (CTAnalyser, SkyScan, 2003–2011; Bruker MicroCT, version 1.12.4.0). Following grayscale calibration and exclusion of non-bone structures, a threshold range between 25 and 90 grayscale levels was applied. This range allowed the accurate quantification of the bone volume within the alveolar sockets. The parameters evaluated included bone volume fraction (BV/TV), trabecular thickness (Tb.Th), trabecular separation (Tb.Sp), trabecular number (Tb.N), and total porosity (Po(tot)) [28].

#### 2.3.6. Analysis and Processing of Decalcified Tissues

Following scanning, the specimens were immersed in a water bath and decalcified in 10% EDTA for 8 weeks. After isolating the alveolar region and adjacent structures, the samples were dehydrated in a graded ethanol series (70–100%), cleared with xylene, and embedded in paraffin. Serial sections (4 μm thick) were obtained from the lingual to vestibular direction, encompassing the alveolus and surrounding tissues. Even-numbered slides were stained with Picrosirius Red and Hematoxylin and Eosin (H&E) for histological analysis.

#### 2.3.7. Microscopic Analysis and Microscopic Area of Interest (AMI)

Microscopic analyses were conducted by a certified histologist blinded to the treatment groups. For histometric evaluation, three histological sections were analyzed per specimen, corresponding to the vestibular, middle, and lingual regions of the extraction site. Following the methodology described by Silva et al. (2024) [28], the first Microscopic Area of Interest (AMI I) encompassed a 4 mm × 4 mm region including the extraction sockets of the mesial and distal roots of the left mandibular first molar and surrounding tissues. The distal limit was defined by a line parallel to the coronal dentin and root of the left mandibular second molar, extending 4 mm mesially. The coronal limit was defined by a line parallel to the cementoenamel junction of the second molar, extending 4 mm apically. The second area (AMI II) included two regions measuring 250 × 250 μm located within the connective tissue above the extraction site. The centers of these areas were defined by a line perpendicular to the tooth axis, dividing the connective tissue in the coronoapical direction. Two additional lines, parallel to the mesial and distal root regions of the extracted first molar, defined the central points of the analyzed zones [28].

#### 2.3.8. Histological Analysis and Inflammatory Profile Evaluation

Qualitative collagen analysis was performed using Picrosirius Red staining. Histological sections were stained with Picrosirius Red according to the manufacturer’s instructions and were examined under polarized light microscopy to evaluate collagen fiber organization, distribution, and maturation. Collagen birefringence was qualitatively assessed based on fiber color and morphology, with green birefringence indicating thin, immature collagen fibers and orange-to-red birefringence indicating thick, mature collagen fibers. Representative photomicrographs were obtained using a Leica DM4000 B LED microscope (Leica Microsystems, Wetzlar, Germany) under standardized magnification and illumination settings [34].

All histological and histometric analyses were performed by a single calibrated and blinded examiner, previously trained for the evaluation criteria, to minimize observer bias and ensure reproducibility of the measurements. Histological analysis, including general tissue characterization and histometric evaluation of the newly formed bone tissue (NFBT), was performed using photomicrographs of the tooth extraction site and adjacent areas. Images were captured using image analysis software (Motic VM 3.0—Motic Digital Slide Assistant, State University of New York, Albany, NY, USA). The NFBT area was measured and expressed in μm^2^ as mean ± standard deviation for each experimental group. In the region of interest (AMI I), image acquisition was conducted using the same software connected to a notebook (IdeaPad 1 AMD, Ryzen 5 7520 U, 8 GB RAM, 512 GB SSD, Windows 11 Home, 15.6″ HD, Brazil). Quantification of the NFBT area and the percentage of non-vital bone tissue (NVBT) was performed using GraphPad Prism version 8.0.0 (GraphPad Software, San Diego, CA, USA). NVBT was defined as regions with more than ten contiguous osteocyte lacunae that were empty or contained remnants of necrotic osteocytes [28].

The inflammatory profile was evaluated using three histological sections per specimen. From each section, three representative photomicrographs were obtained at the center of the extraction socket and in the adjacent right and left regions using a Leica DM4000B light microscope (Leica Microsystems, Wetzlar, Germany) equipped with a DFC500 digital camera and Leica QWin V3 image acquisition software. Images were captured at 100× magnification. The inflammatory infiltrate, with emphasis on mononuclear inflammatory cells, was quantified using the Grid tool in ImageJ (version 1.54d, software (National Institutes of Health, Bethesda, MD, USA)). A standardized grid containing 130 intersection points was superimposed on each image, and inflammatory cells located at the grid intersections were counted. The mean inflammatory cell count for each specimen was used for statistical analysis [28].

#### 2.3.9. Semi-Quantitative Histopathological Evaluation

In addition to the quantitative inflammatory cell count, inflammatory changes were also assessed using a semi-quantitative histopathological scoring system. The intensity and extent of the inflammatory infiltrate were independently evaluated in H&E-stained sections by the blinded examiner according to predefined criteria. The specimens were classified based on the degree of inflammatory cell infiltration and tissue involvement, and the distribution of the scores among the experimental groups was recorded. In addition to the quantitative inflammatory cell count, a semi-quantitative histopathological evaluation of the inflammatory response was performed on H&E-stained sections by the same blinded and calibrated examiner. The inflammatory response was scored according to the intensity and extent of the inflammatory infiltrate. The intensity of inflammation was classified as: (1) absence of inflammation; (2) moderate number of inflammatory cells; and (3) severe number of inflammatory cells. The extent of inflammation was classified as: (1) absence of inflammation; (2) partial extension within the connective tissue; (3) full extension throughout the connective tissue without reaching the bone tissue; and (4) full extension throughout the connective and bone tissues. The distribution of the scores was recorded for each experimental group and used to characterize the inflammatory profile [13].

#### 2.3.10. Immunohistochemical Evaluation

Immunohistochemistry was performed on semiserial sections collected at each euthanasia time point using the immunoperoxidase method. Endogenous peroxidase was inhibited with hydrogen peroxide, followed by antigen retrieval with a citrate–phosphate buffer (pH 6.0) and biotin blocking with skim milk. The primary antibodies used were anti-VEGF and anti-osteocalcin (Santa Cruz Biotechnology Dallas, TX, USA). A biotinylated anti-goat secondary antibody (Jackson ImmunoResearch Laboratories, West Grove, PA, USA). and the Avidin-Biotin Complex (Vector Laboratories, Newark, CA, USA) were applied, with DAB (Dako, Glostrup, Denmark) as the chromogen. Sections were counterstained with Harris Hematoxylin. Protein expression was semi-quantitatively assessed based on the number of immunolabeled cells involved in bone repair, using a light microscope (Leica^®^ DMLB, Heerbrugg, Switzerland). Immunolabeling intensity was scored from 1 (no labeling) to 4 (intense labeling).

#### 2.3.11. Statistical Analysis

All data were first tested for normality using the Shapiro–Wilk test, followed by one-way ANOVA and Tukey’s post hoc test (*p* < 0.05). The statistical software used was GraphPad Prism version 8.0.0 (GraphPad Software, San Diego, CA, USA).

## 3. Results

### 3.1. Condition Assessment of the Animals and Extraoral and Intraoral Examination

**Figure 2:**
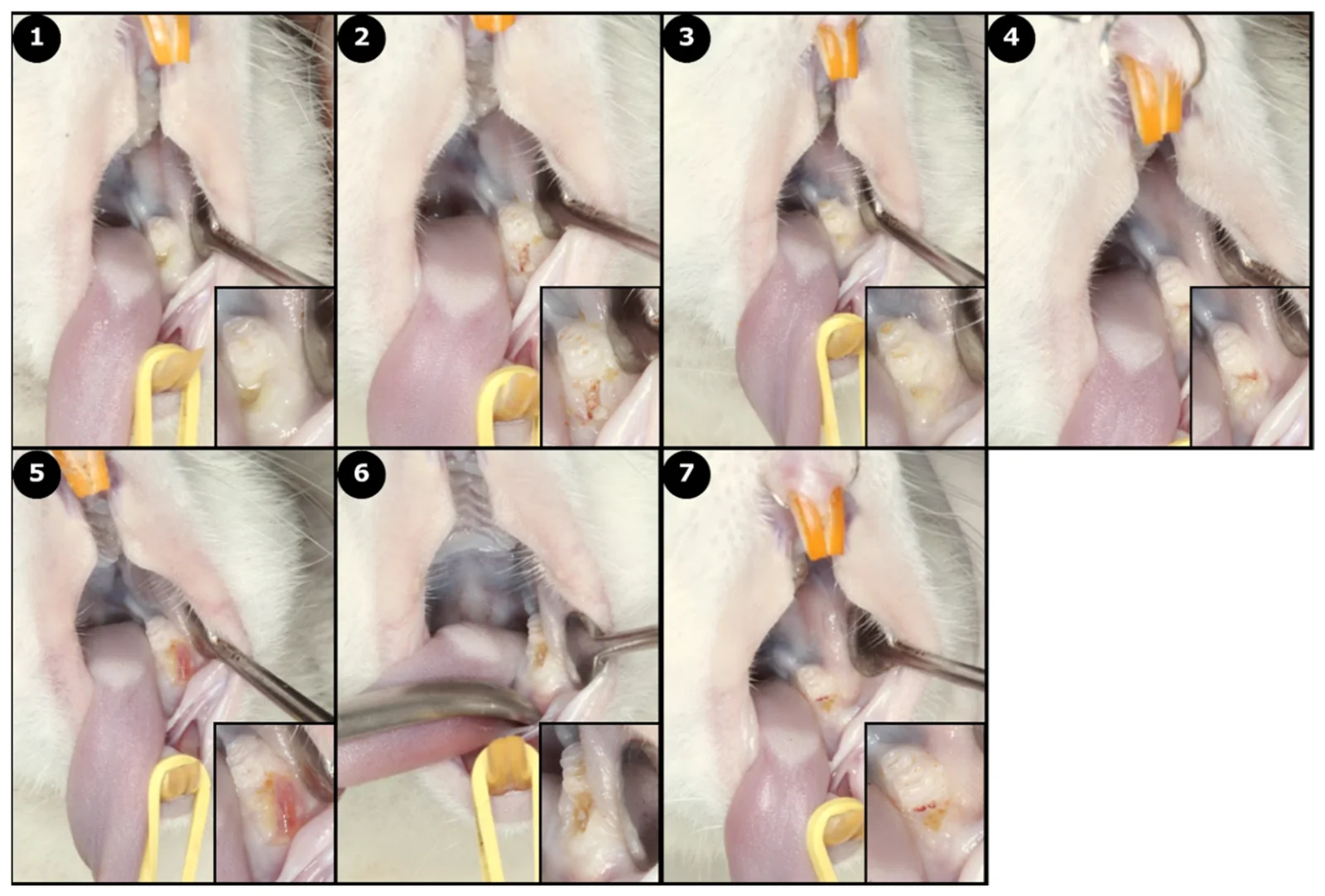
Clinical photographs of the animals, including extraoral and intraoral examinations performed prior to euthanasia. (**1**) Saline group; (**2**) zoledronate group (Zol); (**3**) ozone group; (**4**) MTZ group; (**5**) MTZ + ozone group; (**6**) CHX group; (**7**) CHX + ozone group. Before sample collection, the clinical appearance of the operated region was documented using standardized photographs captured by the same operator with a Nikon D7000 digital camera equipped with a 105 mm macro lens and a macro flash.

The general health of the animals remained stable throughout the experimental period. All animals tolerated the first molar extraction procedure and, when applicable, the therapeutic interventions in the ZOL+OZ, ZOL+MTZ, ZOL+CLX, ZOL+MTZ+OZ, and ZOL+CLX+OZ groups. At the end of the study, no statistically significant differences in mean body weight were observed among the groups: SAL (364 ± 7 g), ZOL (350 ± 7 g), ZOL+OZ (350 ± 5 g), ZOL+MTZ (346 ± 7 g), ZOL+CLX (342 ± 7 g), ZOL+MTZ+OZ (350 ± 7 g), and ZOL+CLX+OZ (347 ± 7 g). Likewise, no significant intra-group weight variation was observed over time. Representative intraoral photographs demonstrated complete soft tissue coverage of the extraction socket in the SAL and ZOL+OZ groups, whereas the ZOL group exhibited persistent bone exposure and discontinuity of the overlying soft tissue. In the remaining treatment groups, localized areas of bone exposure were still observed, although the surrounding soft tissues appeared more continuous than those observed in the ZOL group. As no predefined clinical scoring system was used, these findings are presented as representative qualitative observations only (Figure 2).

### 3.2. Micro-CT Analysis

Regarding bone volume fraction (BV/TV), the SAL group showed a statistically significant difference compared with all other groups (*p* < 0.001). The OZ group also differed significantly from the MTZ (*p* = 0.002) and CLX (*p* < 0.0013) groups. No statistically significant differences were observed among the MTZ, MTZ+OZ, CLX, and CLX+OZ groups. For trabecular thickness (Tb.Th), the SAL group exhibited lower trabecular thickness than the ZOL group (*p* < 0.001). However, no statistically significant differences were observed between the SAL group and the remaining experimental groups.

**Figure 3:**
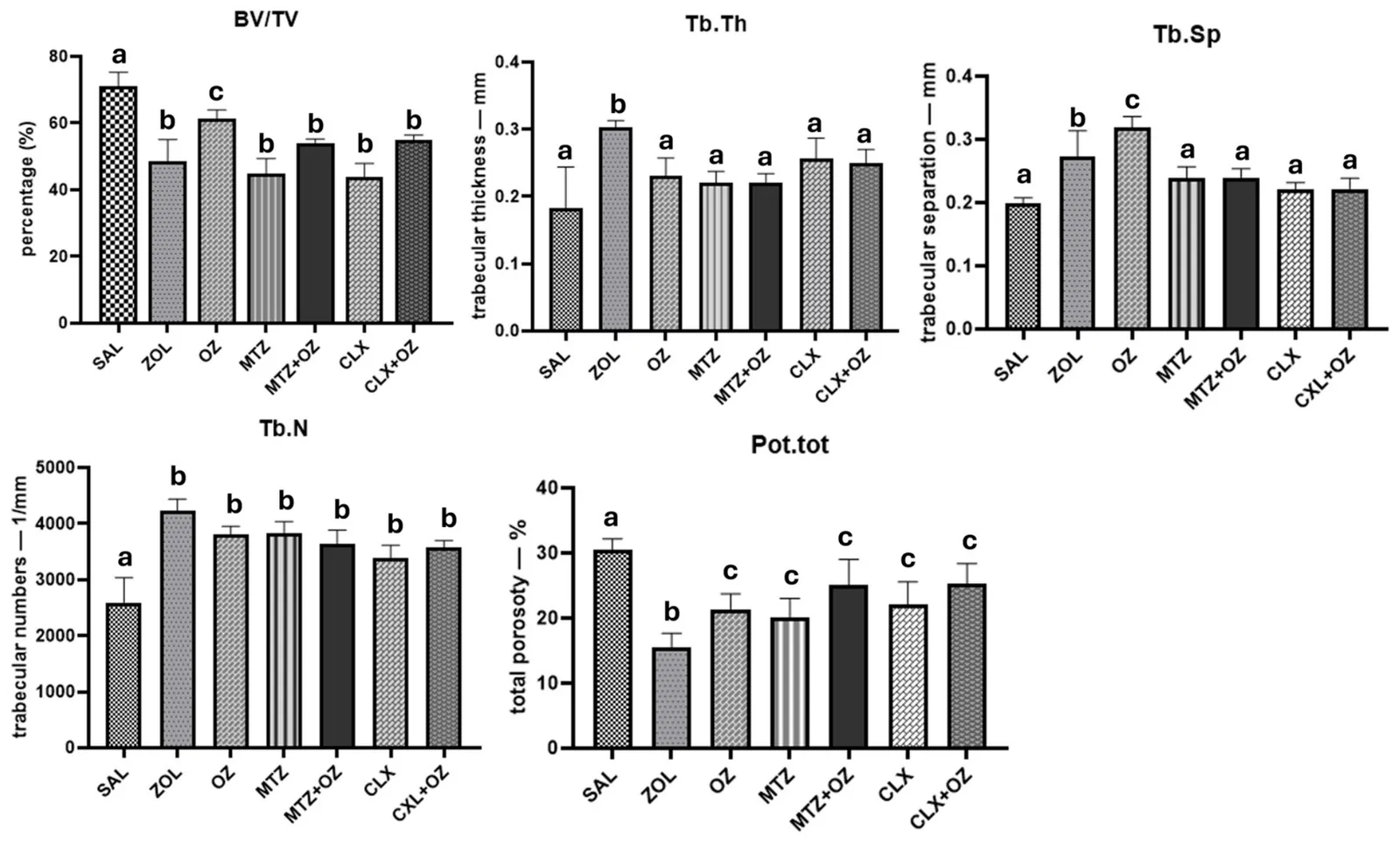
Graphs showing the mean ± standard deviation of the micro-CT parameters BV/TV, Tb.Th, Tb.Sp, Tb.N, and Po(tot) at the 60-day time point for the SAL, ZOL, ZOL+OZONE, ZOL+OZ+MTZ, ZOL+MTZ, ZOL+OZ+CLX, and ZOL+CLX groups. Lowercase letters indicate statistically significant intergroup differences (*p* < 0.05).

Regarding trabecular separation (Tb.Sp), the SAL group differed significantly from the ZOL and OZ groups (*p* < 0.001), whereas no significant differences were observed in comparison with the remaining groups. The OZ group, in turn, showed statistically significant differences compared with all other groups. With respect to trabecular number (Tb.N), the SAL group differed significantly from all other groups (*p* < 0.001), whereas no statistically significant differences were observed among the remaining experimental groups. Regarding total bone porosity Po(tot), the SAL group exhibited the highest values compared with all other groups (*p* < 0.001), whereas the ZOL group presented the lowest bone porosity. The groups treated with ozone therapy, either alone or in combination with adjunctive therapies, showed significantly higher bone porosity than the ZOL group (Figure 3).

### 3.3. Histological and Histometric Analysis

#### 3.3.1. Histopathological Analysis and Picrosirius Red Staining

Histological analysis of the SAL group revealed well-organized epithelial tissue, with tightly adherent cells forming continuous layers over the buccal gingiva of the extraction site. The underlying connective tissue appeared normal, consisting of loose lamina propria with vascular structures. In contrast, the ZOL group exhibited pronounced morphological alterations, including epithelial invaginations and thickening, as well as denser connective tissue with increased cellularity and vascularity, indicative of inflammation. The OZ group also showed epithelial invaginations and thickening but appeared more organized than the ZOL group, with dense, yet less inflamed, connective tissue.

**Figure 4:**
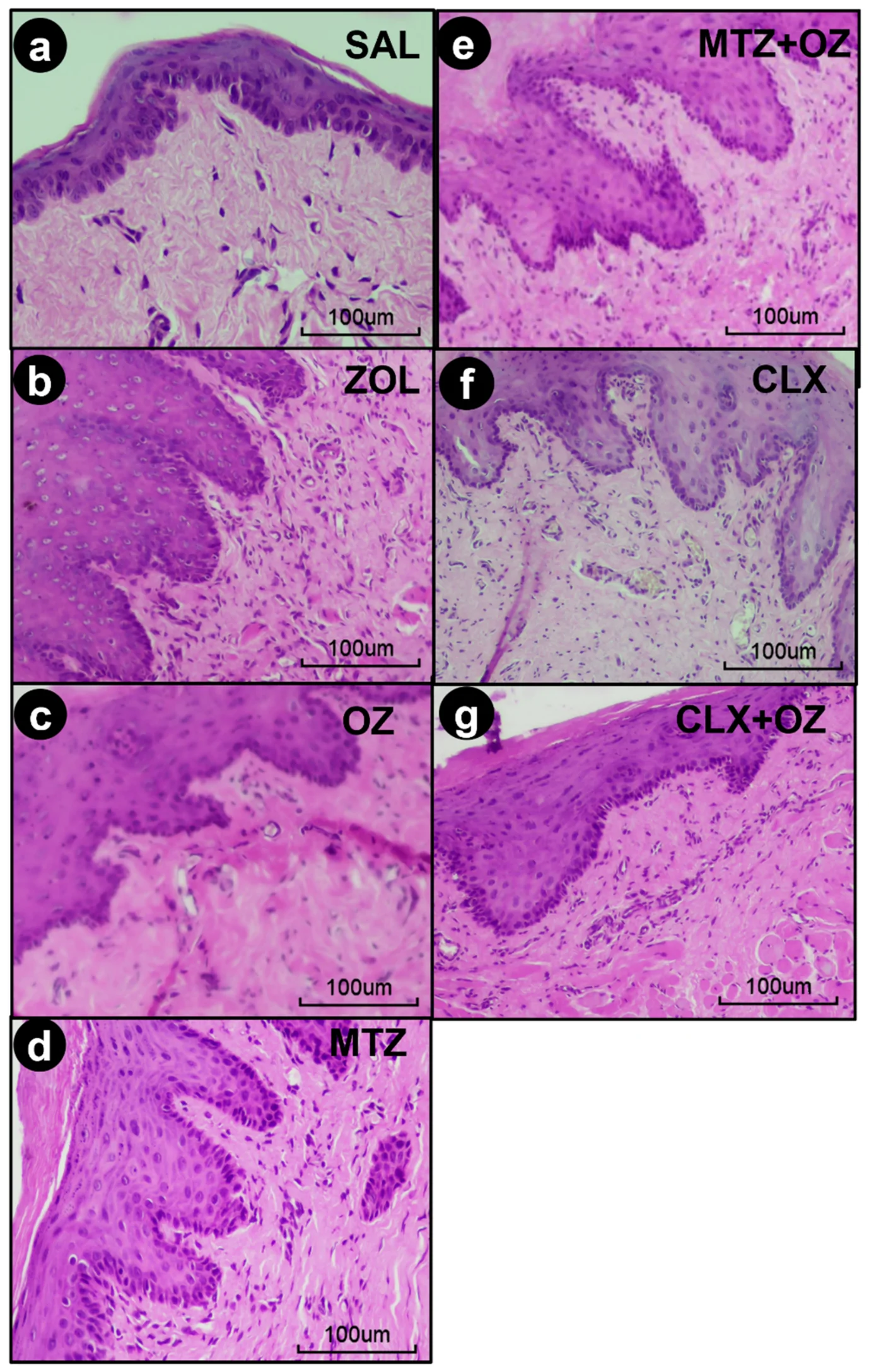
Histological appearance of the vestibular epithelium and connective tissue collected at 28 days postoperatively. (**a**–**g**) Photomicrographs showing the histological features of the epithelial and connective tissue at the tooth extraction site at 28 days postoperatively in SAL (**a**), ZOL (**b**), OZ (**c**), MTZ (**d**), MTZ+OZ (**e**), CLX (**f**), and CLX+OZ (**g**) groups. Original magnification: 100×. Scale bar: 100 µm. Staining: Hematoxylin and Eosin (H&E).

In the MTZ group, more pronounced epithelial invaginations and thickening were observed compared to the MTZ+OZ group. Both groups exhibited dense and structured connective tissue; however, the MTZ+OZ group had a higher vascular density. The CLX group demonstrated non-keratinized stratified squamous epithelium with marked morphological changes, including deep epithelial invaginations extending into the connective tissue. In the CLX+OZ group, although invaginations were present, epithelial architecture was notably more preserved compared to CLX alone. The connective tissue remained dense in both groups, with a higher number of blood vessels observed in the CLX group (Figure 4).

**Figure 5:**
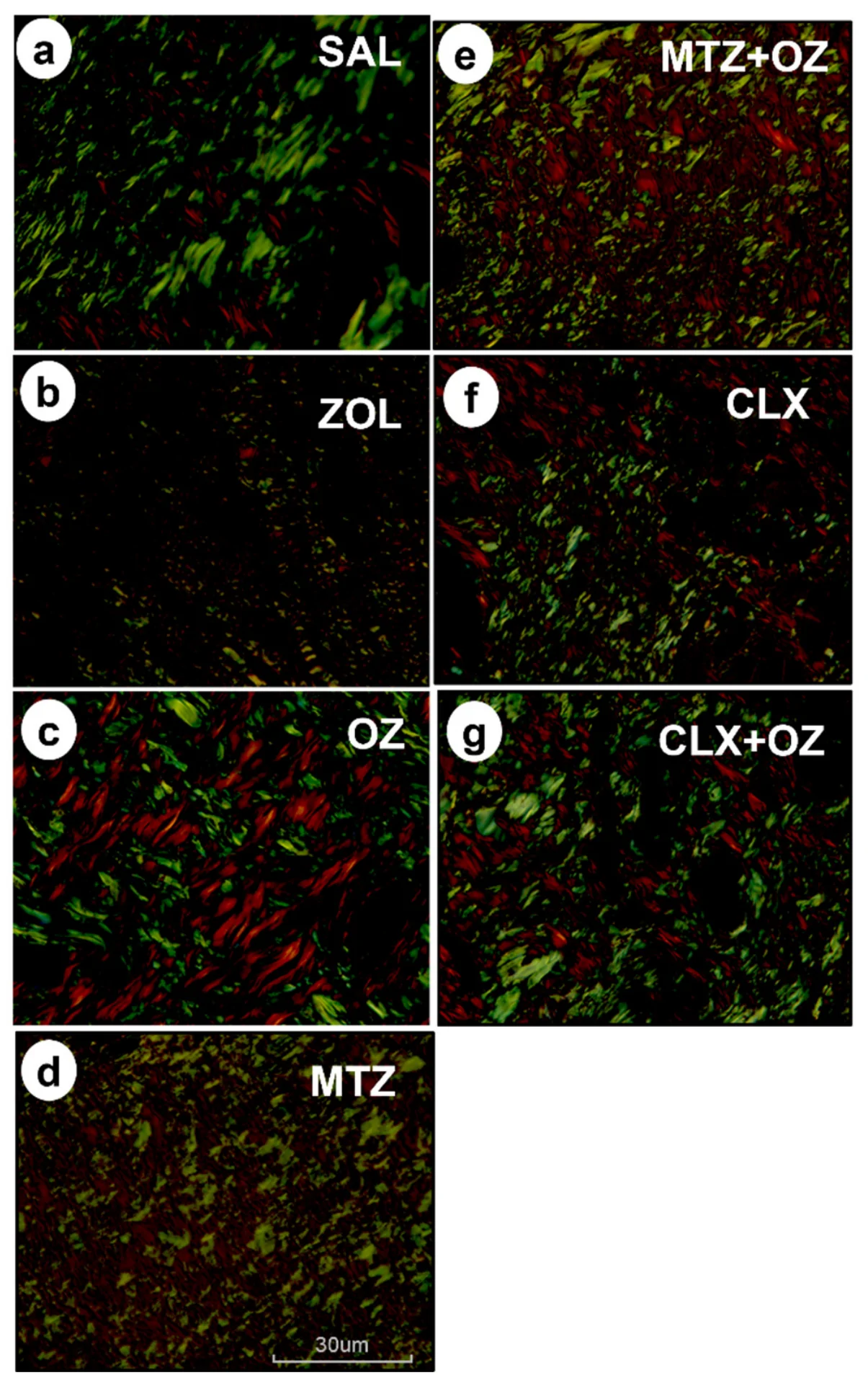
Representative images of Picrosirius Red (PSR) staining for the evaluation of collagen fiber maturity. (**a**) SAL group; (**b**) ZOL group; (**c**) ZOL+OZ group; (**d**) MTZ group; (**e**) MTZ+OZ group; (**f**) CLX group; (**g**) CLX+OZ group. PSR staining; original magnification, ×300.

Qualitative analysis of Picrosirius Red-stained sections under polarized light revealed differences in collagen fiber birefringence and organization among the experimental groups. The SAL group exhibited a mixed pattern of green and red birefringence, consistent with the presence of both immature and mature collagen fibers, suggesting ongoing extracellular matrix remodeling. In contrast, the ZOL group showed sparse collagen birefringence with limited evidence of organized collagen fibers. The OZ, MTZ+OZ, and CLX+OZ groups displayed areas containing both green and red birefringent fibers, indicating the coexistence of immature and mature collagen within the connective tissue. Similarly, the CLX and MTZ groups exhibited mixed collagen birefringence, with mature collagen fibers interspersed with green birefringent fibers. As this evaluation was qualitative, no statistical comparisons were performed (Figure 5).

#### 3.3.2. Histometric Analysis of the Gingiva

Statistical analysis of maximal epithelial thickness revealed that the SAL group differed significantly from all other groups, followed by the ZOL group (*p* < 0.0001). No significant differences were observed between the OZ group and the MTZ (*p* = 0.547) or CLX (*p* = 0.988) groups; however, the OZ group differed significantly from the MTZ+OZ and CLX+OZ groups (*p* < 0.001). The MTZ+OZ and CLX+OZ groups did not differ from each other (*p* = 0.994).

**Figure 6:**
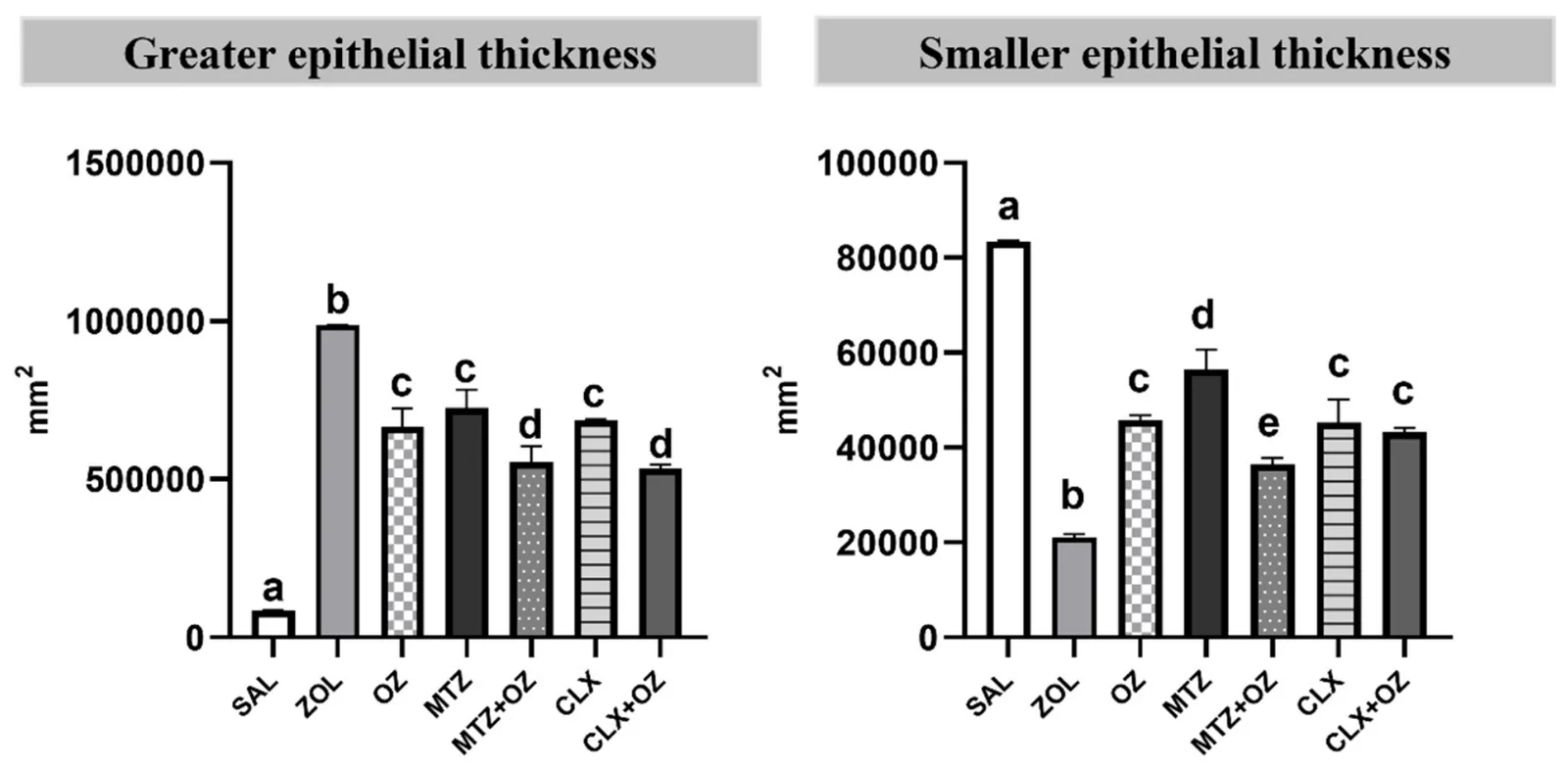
Analysis of the maximum and minimum thickness of the gingival epithelium at the dental extraction site 28 days post-exodontia. Statistical tests: Shapiro–Wilk, Analysis of Variance (ANOVA), and Tukey’s post hoc test. Lowercase letters indicate statistically significant differences between groups.

For minimal epithelial thickness, the SAL group exhibited the greatest difference compared to other groups (*p* < 0.001), indicating a thinner epithelium. The ZOL group also differed significantly from the others (*p* < 0.001), suggesting fewer regions with reduced epithelial thickness. The OZ group showed no significant difference compared to CLX (*p* > 0.999) and CLX+OZ (*p* = 0.937) but differed from the MTZ+OZ group (*p* < 0.001). The MTZ group differed significantly from all other groups, including controls and test groups (*p* < 0.001) (Figure 6).

#### 3.3.3. Histometric Analysis

**Figure 7:**
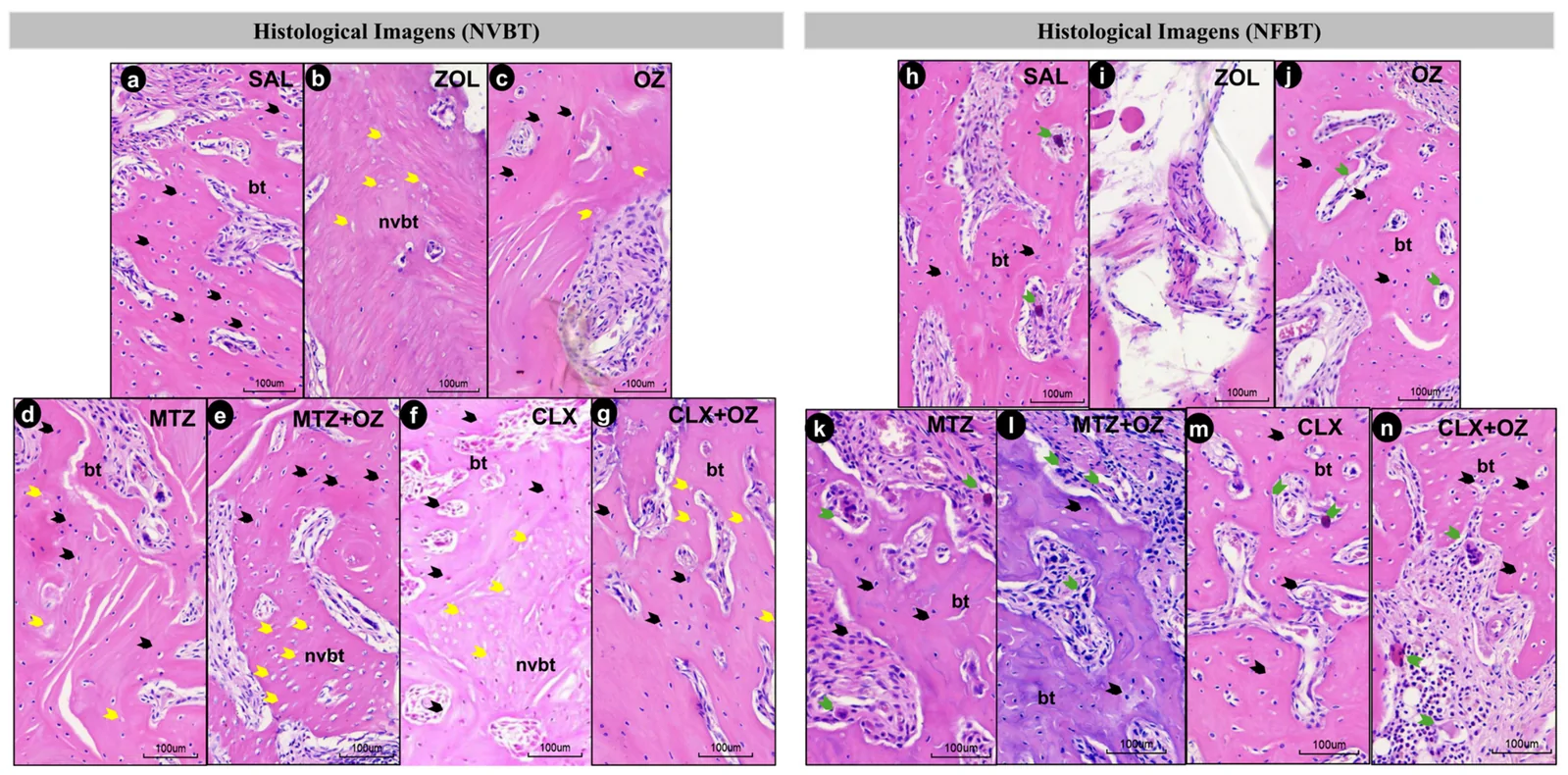
Photomicrographs depicting histological features of bone and connective tissues at the tooth extraction site 28 days postoperatively in SAL (**a**,**h**), ZOL (**b**,**i**), OZ (**c**,**j**), MTZ (**d**,**k**), MTZ+OZ (**e**,**l**), CLX (**f**,**m**), and CLX+OZ (**g**,**n**). Panels (**a**–**g**) highlight green arrows, osteoclasts; black arrows, osteoblasts; bt, bone tissue. Panels highlight yellow arrows, empty lacunae; nvbt, non-vital bone tissue; black arrows, osteoblasts; bt, bone tissue. Original magnification: 60×; scale bars = 100 µm. Staining: Hematoxylin and Eosin (H&E).

**Figure 8:**
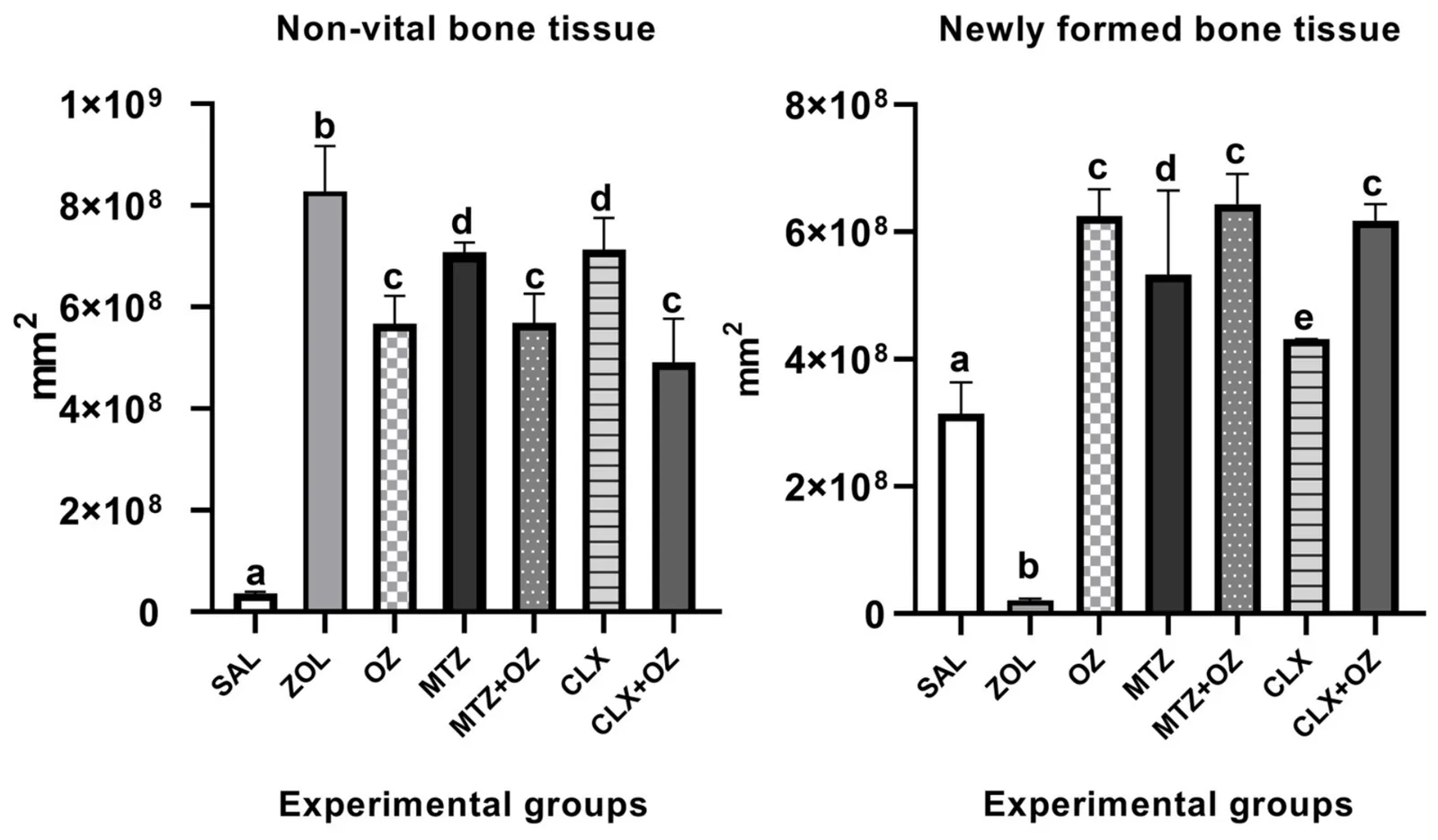
Graphs represent newly formed bone tissue (NFBT) and non-vital bone tissue (NVBT). Statistical analyses were performed using Shapiro–Wilk, ANOVA, and Tukey’s post hoc tests. Lowercase letters denote statistically significant differences between groups.

Necrotic tissue was observed in adjacent regions of the ZOL group, with the alveolar interior appearing largely empty. In contrast, groups receiving adjuvant therapies—alone or in combination—exhibited substantial areas of vital bone containing osteocyte-filled lacunae and minimal necrosis. Analysis of newly formed bone (NFB) area revealed that the ZOL group had the smallest NFB compared to all other groups (*p* < 0.001). The SAL group showed statistically greater bone formation than the ZOL, OZ, MTZ, MTZ+OZ, and CLX+OZ groups (*p* < 0.001). The OZ group did not differ significantly from MTZ (*p* = 0.393), MTZ+OZ (*p* = 0.999), or CLX+OZ (*p* = 0.999), but differed from CLX (*p* < 0.001). The MTZ group showed no significant difference compared to MTZ+OZ (*p* = 0.207), CLX (*p* = 0.361), or CLX+OZ (*p* = 0.566). The CLX group differed significantly from CLX+OZ (*p* = 0.021), as well as from OZ and ZOL (*p* < 0.001). No significant difference was found between MTZ+OZ and CLX+OZ groups (*p* = 0.997) (Figure 7 and Figure 8).

Analyzing the area of non-vital bone tissue (NVB area), the ZOL group (*p* < 0.05) showed a greater result compared to the other therapies, followed by the MTZ (*p* = 0.125) and CLX (*p* = 0.152) groups. The OZ group did not present significant statistical differences when compared to the treatment groups MTZ, MTZ+OZ, CLX, and CLX+OZ (*p* < 0.001). The groups that did not receive ozone therapy, MTZ and CLX, showed no statistical differences between each other (*p* < 0.001) (Figure 7 and Figure 8). The semi-quantitative histopathological analysis corroborated these findings. The ZOL group exhibited the highest frequency of moderate-to-severe inflammatory infiltrate and the greatest extension of inflammation into the connective and bone tissues, whereas the SAL group showed no evidence of inflammation. In contrast, the adjuvant therapy groups were predominantly characterized by the absence of inflammation or only moderate inflammatory infiltrate, with limited extension restricted to the connective tissue (Table 1 and Table 2).

#### 3.3.4. Inflammatory Profile Analysis

**Figure 9:**
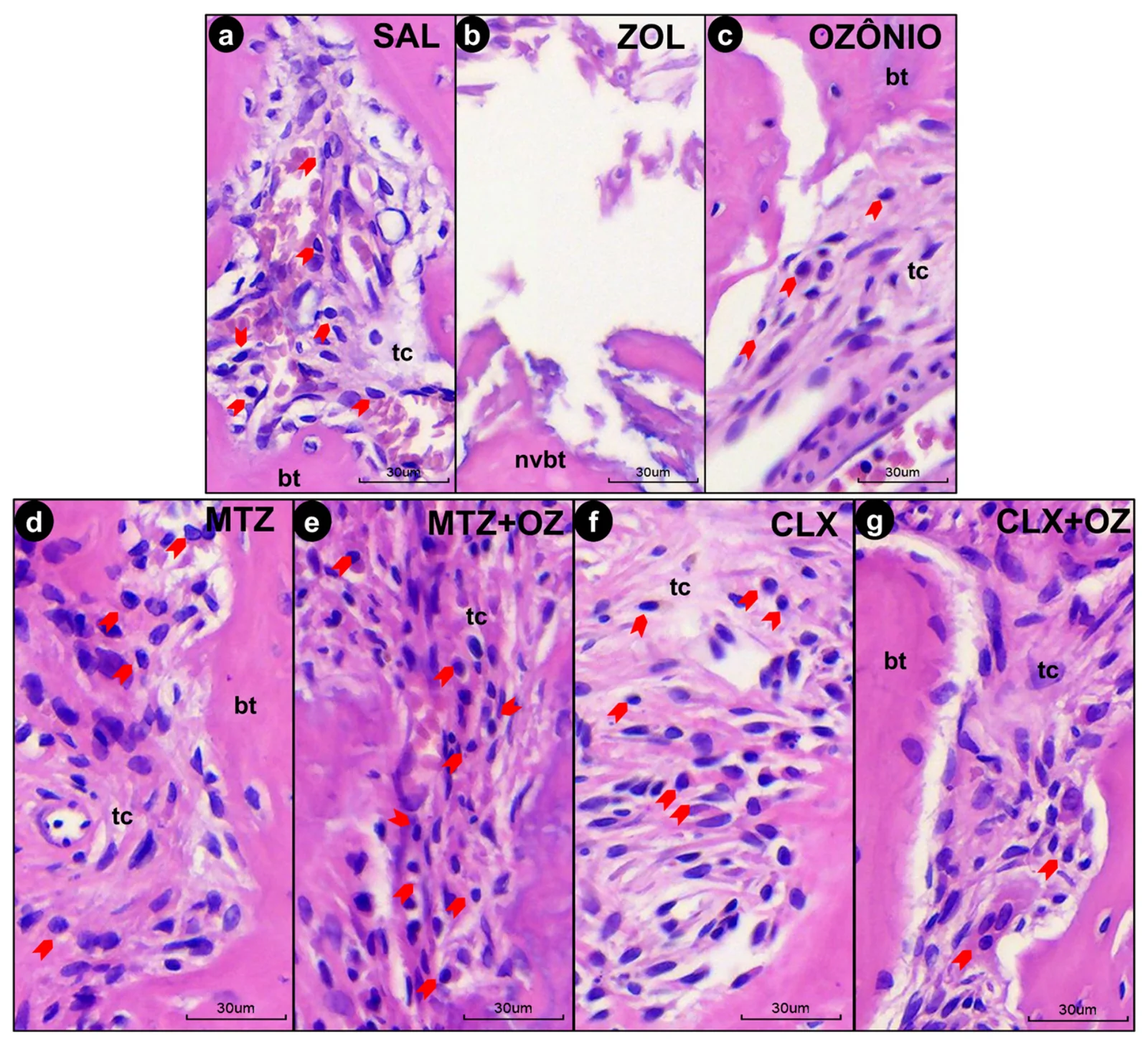
Inflammatory profile at the dental extraction site 28 days postoperatively. (**a**–**g**) Photomicrographs showing histological features of bone and connective tissues in SAL (**a**), ZOL (**b**), OZ (**c**), MTZ (**d**), MTZ+OZ (**e**), CLX (**f**), and CLX+OZ (**g**) groups. Abbreviations and symbols: the arrows indicate inflammatory cells. Original magnification: 400×; scale bars = 100 µm. Staining: Hematoxylin and Eosin (H&E).

**Figure 10:**
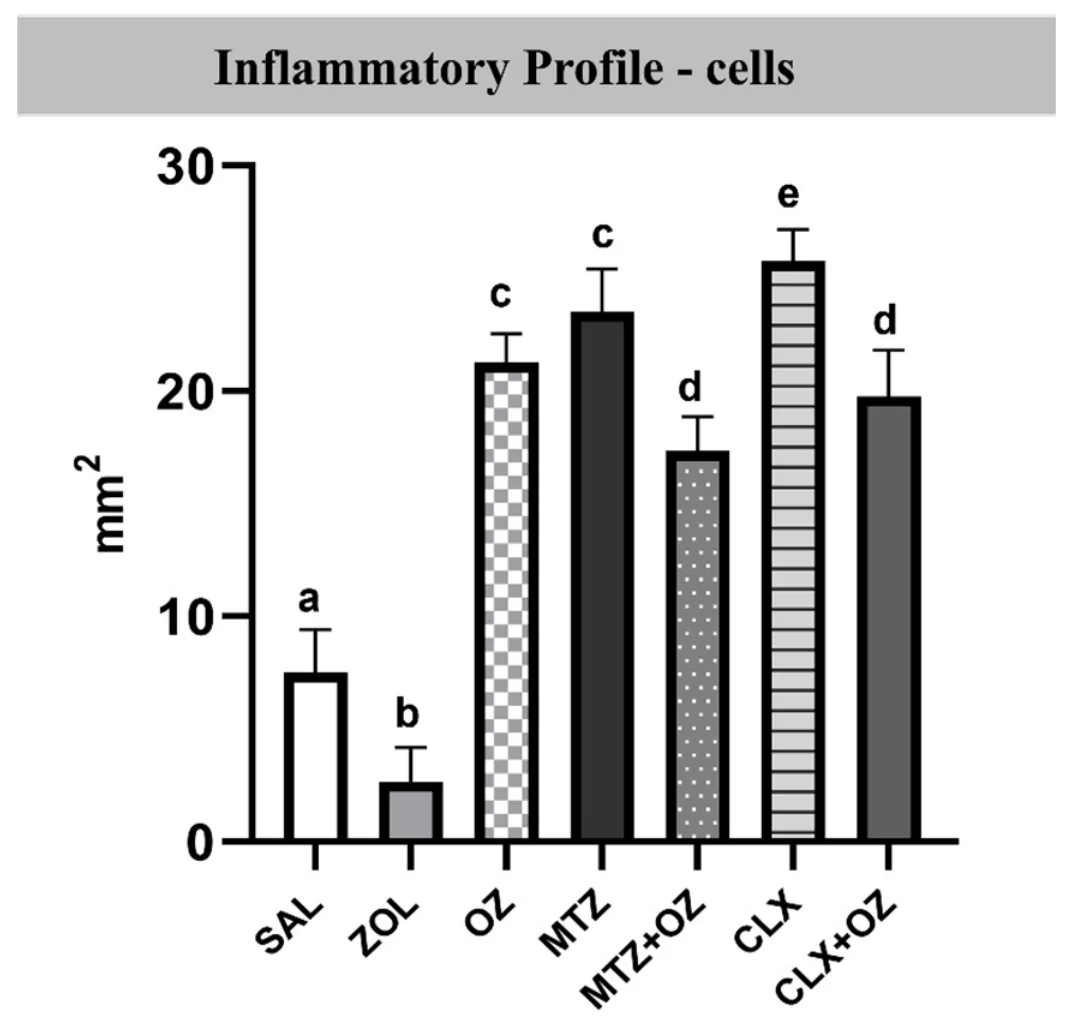
Inflammatory profile of the tooth extraction site at 28 days post-extraction. Statistical tests: Shapiro–Wilk, Analysis of Variance (ANOVA), and Tukey’s post-test. Lowercase letters indicate statistical differences between groups.

In the inflammatory profile analysis, the SAL group demonstrated a statistically significant difference compared to all other groups (*p* < 0.001). Although the ZOL group exhibited the lowest inflammatory cell count (*p* < 0.001), histological examination revealed that the extraction sockets were largely devoid of cellular and connective tissue components due to extensive tissue necrosis, resulting in an almost empty alveolar space. Therefore, the reduced inflammatory cell count in this group should not be interpreted as a lower inflammatory response, but rather as the absence of viable tissue available for evaluation. The OZ group did not differ statistically from the MTZ group (*p* = 0.509). The CLX group showed statistically significant differences compared with all other groups (*p* < 0.001). In contrast, the MTZ+OZ and CLX+OZ groups were statistically similar (*p* = 0). Histological analysis showed that the treatment groups exhibited dense connective tissue, whereas the SAL group displayed more disorganized connective tissue with a greater number of blood vessels (Figure 9 and Figure 10).

### 3.4. Immunohistochemical Analysis

#### 3.4.1. VEGF Analysis

**Figure 11:**
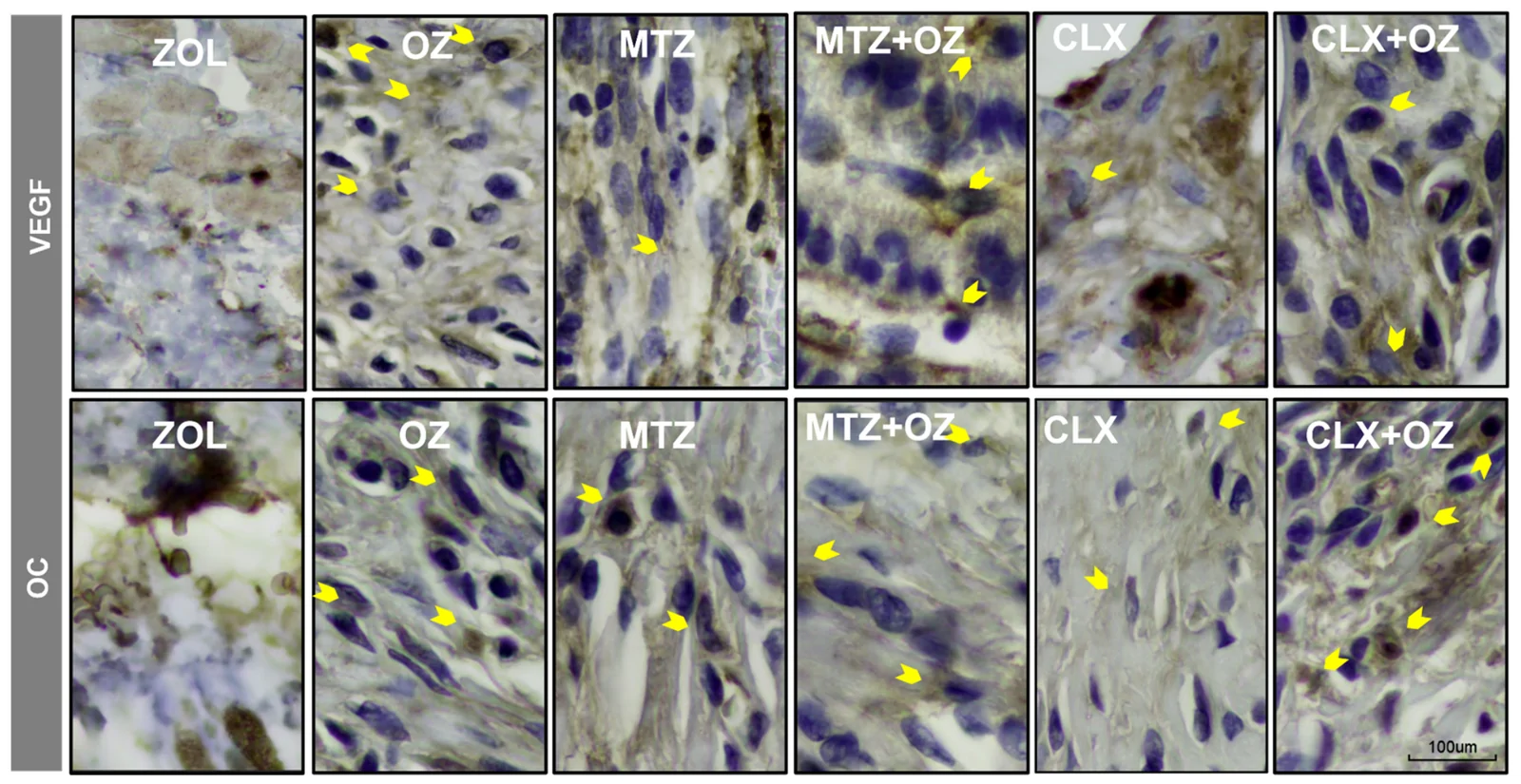
Immunohistochemical analysis of the dental extraction site at 28 days postoperatively. Photomicrographs showing VEGF and osteocalcin (OC) markers. Yellow arrows indicate immunopositive cells. Original magnification: 1000×. Scale bars: 100 µm.

In a semi-quantitative comparison, the MTZ+OZ and OZ groups showed higher immunostaining compared to the other experimental groups. The ZOL group did not show immunostaining. Groups that received adjuvant therapies without association with ozone exhibited a low immunostaining pattern. The CLX+OZ group presented a moderate immunostaining pattern (Figure 11).

#### 3.4.2. Osteocalcin Analysis

In a semi-quantitative comparison, the MTZ+OZ and CLX+OZ groups exhibited higher immunostaining compared to the other experimental groups. The ZOL group showed no immunostaining. The groups that received adjuvant therapies without ozone association demonstrated a moderate immunostaining pattern (Figure 11).

## 4. Discussion

The present study investigated the effects of systemic ozone therapy, alone and combined with topical antimicrobial agents, on the prevention of medication-related osteonecrosis of the jaws (MRONJ) in an experimental model of zoledronate-induced alveolar repair impairment. Overall, the findings demonstrated that ozone therapy, particularly when administered alone or in combination with topical metronidazole or chlorhexidine, promoted more favorable tissue repair than zoledronate treatment alone, as evidenced by histological, histomorphometric, micro-computed tomography, and collagen birefringence analyses [13]. As bisphosphonates (BPs) reduce bone remodeling by acting on osteoclasts and osteoblasts, alterations in bone quality and quantity were expected [35].

The animals treated exclusively with zoledronate exhibited the most severe impairment of alveolar repair. Histomorphometric analysis demonstrated a marked reduction in newly formed bone and a significant increase in non-vital bone tissue [23,35]. Histologically, extraction sockets were largely devoid of reparative connective tissue and showed pronounced epithelial disorganization, including thinning and irregularity of the superficial keratin layer, suggesting impaired epithelial maturation and barrier function. These findings are consistent with the mechanism of action of nitrogen-containing bisphosphonates, which inhibit the mevalonate pathway, preventing activation of proteins required for osteoclast function and leading to a profound suppression of bone remodeling [36,37,38]. Although this pharmacological effect is essential for controlling osteometabolic and neoplastic diseases, prolonged suppression of osteoclastic activity compromises physiological bone turnover, preventing damaged bone and deposition of the newly formed matrix, resulting in the accumulation of devitalized mineralized tissue, a characteristic feature of MRONJ reported in both experimental models and clinical studies [35,39].

The micro-computed tomography findings further support this interpretation. Although the zoledronate-treated animals exhibited greater trabecular thickness and the lowest total bone porosity, these findings should not be interpreted as evidence of superior bone quality. Instead, they likely reflect the accumulation of dense, sclerotic bone resulting from the profound suppression of bone turnover, a well-established feature of bisphosphonate-associated osteonecrosis. In contrast, ozone-treated animals showed a higher bone volume fraction than animals receiving topical therapies alone. Moreover, ozone administration, either alone or combined with topical treatments, resulted in higher total bone porosity than untreated zoledronate animals. Considering the profound suppression of bone turnover induced by zoledronate, these findings may indicate partial restoration of physiological bone remodeling rather than deterioration of bone quality. When considering the histological evidence of enhanced bone formation and improved tissue organization, these observations reinforce the regenerative potential of ozone therapy [28,40].

Alveolar repair differs substantially from healing at other skeletal sites as it relies on the coordinated interaction among bone remodeling, connective tissue maturation, epithelial closure, and local inflammatory regulation following tooth extraction [40,41]. Therefore, therapeutic strategies capable of modulating these biological processes may contribute to reducing the severity of MRONJ lesions. Although no universally accepted treatment protocol currently exists, conservative adjunctive therapies have been increasingly recommended, particularly during the early stages of the disease. These interventions aim to preserve tissue integrity, minimize symptoms, and improve patients’ quality of life [26,38].

Among the therapies investigated, systemic ozone therapy demonstrated the most consistent regenerative profile. Experimental evidence indicates that ozone promotes controlled oxidative preconditioning, stimulating endogenous antioxidant defenses, modulating inflammatory mediators, improving microcirculation, and favoring tissue repair [17,27,42,43,44]. Importantly, systemic ozone administration produced no detectable adverse effects on liver, kidney, or lung tissues, corroborating with previous experimental studies demonstrating its biological safety [28,41]. Histologically, ozone-treated animals exhibited greater cellular organization and significantly higher new bone formation than untreated zoledronate animals. These findings are consistent with previous reports describing beneficial effects of ozone therapy on bone repair.

The qualitative Picrosirius Red analysis demonstrated differences in collagen fiber organization among the experimental groups. Whereas the zoledronate group exhibited sparse and poorly organized collagen birefringence, ozone-treated groups showed the coexistence of green and red birefringent collagen fibers, consistent with active extracellular matrix remodeling during tissue repair. Because this analysis was qualitative, these findings should be interpreted as descriptive observations rather than quantitative evidence of collagen maturation.

The present study also evaluated two topical antimicrobial therapies commonly employed in oral wound management. Metronidazole paste and 2% chlorhexidine gel have been proposed as local therapeutic alternatives because they provide antimicrobial activity while avoiding several disadvantages associated with prolonged systemic antibiotic therapy, including bacterial resistance, adverse reactions, and drug interactions [45,46,47]. Histologically, both therapies were associated with improved tissue organization compared with the untreated zoledronate group. Metronidazole-treated animals showed increased formation of vital bone tissue and reduced areas of osteonecrosis, whereas chlorhexidine-treated animals demonstrated comparable improvements in connective tissue repair. However, because no microbiological analyses were performed, the present findings should not be interpreted as direct evidence of infection control, but rather as histological tissue responses following topical treatment. The combination of systemic ozone therapy with topical metronidazole or chlorhexidine also resulted in favorable histological findings. Animals receiving combined therapies exhibited improved epithelial organization, connective tissue maturation, and bone repair compared with untreated zoledronate-exposed animals. Nevertheless, although these combined treatments produced better outcomes than zoledronate alone, the present study was not designed to evaluate statistical interaction between ozone and topical therapies. Therefore, the observed improvements should be interpreted as comparative findings among experimental groups rather than evidence of synergistic or potentiating effects.

The interpretation of the immunohistochemical findings also deserves caution. The increased VEGF and osteocalcin immunostaining observed in ozone-treated groups is consistent with an enhanced expression of angiogenic- and osteogenic-related markers during tissue repair. However, because functional angiogenesis, osteoblast maturation, tissue perfusion, or molecular signaling pathways were not directly evaluated, these findings should be interpreted as indirect evidence of biological activity rather than proof of enhanced angiogenesis or osteoblast differentiation. Some limitations should be acknowledged. The study evaluated a single experimental period following tooth extraction, microbiological analyses were not performed, collagen evaluation was qualitative, and the immunohistochemical analysis was limited to selected biomarkers. Furthermore, the experimental protocol included a limited number of ozone applications, and only one route of systemic administration was investigated. Future studies incorporating longitudinal evaluation, quantitative collagen analysis, molecular markers, and microbiological assessment may further clarify the biological mechanisms underlying the beneficial effects observed.

Within the limitations of this study, the present findings indicate that systemic ozone therapy, particularly when associated with topical antimicrobial treatment, improved several histological and microstructural parameters of alveolar repair in zoledronate-treated animals. These results support the potential of conservative adjunctive therapies as promising strategies to enhance tissue repair during the early stages of MRONJ. However, further preclinical and clinical studies are required before their incorporation into routine clinical practice.

## 5. Conclusions

Systemic ozone therapy improved histological and microstructural parameters of alveolar repair in zoledronate-treated rats. Topical application of 10% metronidazole paste and 2% chlorhexidine gel also resulted in favorable tissue repair findings. Moreover, the combined therapy showed more favorable outcomes than zoledronate treatment alone, supporting the potential of these conservative approaches as adjunctive strategies for the prevention and management of medication-related osteonecrosis of the jaws. Further preclinical and clinical studies are warranted to confirm these findings.

## Figures and Tables

**Table 1:** Scores and distribution of the specimens according to the histopathological analysis of the level of tissue inflammation during the repair process after dental extraction in SAL, ZOL, OZONE, MTZ, MTZ+OZ, CLX, and CLX+OZ groups at 28 days postoperatively.

Histopathological Analysis: Tissue Structure Pattern	Experimental Groups Number of Specimens						
	*SAL* (*n* = 6)	*ZOL* (*n* = 6)	*OZ* (*n* = 6)	*MTZ* (*n* = 6)	*MTZ+OZ* (*n* = 6)	*CLX* (*n* = 6)	*CLX+OZ* (*n* = 6)
*Cellular Organization and Epithelial Tissue Structure*							
(1) Absence of inflammation	6	0	4	3	4	3	4
(2) Moderate number of inflammatory cells	0	2	2	3	1	2	2
(3) Severe number of inflammatory cells	0	4	0	0	1	1	1
*Inflammation Extent*							
(1) Absence of inflammation	6	0	4	4	3	4	2
(2) Partial extension within the connective tissue	0	0	2	2	3	2	4
(3) Full extension throughout the connective tissue without reaching the bone tissue	0	3	0	0	0	0	0
(4) Full extension throughout the connective and bone tissues	0	3	0	0	0	0	0

**Table 2:** Scores and distribution of the specimens according to the histopathological analysis of the level of tissue. Inflammation during the repair process after dental extraction in the SAL, ZOL, OZONE, MTZ, MTZ+OZ, CLX and CLX+OZ groups at 28 days postoperatively.

Histopathological Analysis: Tissue Structure Pattern	Experimental Groups Number of Specimens						
	*SAL* (*n* = 6)	*ZOL* (*n* = 6)	*OZ* (*n* = 6)	*MTZ* (*n* = 6)	*MTZ+OZ* (*n* = 6)	*CLX* (*n* = 6)	*CLX+OZ* (*n* = 6)
*Cellular Architecture and Epithelial Tissue Structure*							
(1) Moderate thickness epithelial tissue completely covering the extraction site	2	0	5	3	4	3	3
(2) Thin epithelial tissue completely covering the extraction site	4	2	1	3	2	3	3
(3) Thin layer of epithelial tissue only at the edges of the surgical wound	0	4	0	0	0	0	0
(4) Absence of epithelial tissue in the open surgical wound	0	0	0	0	0	0	0
*Cellular Architecture and Connective Tissue Structure*							
(1) Moderate number of fibroblasts and large amount of collagen fibers	5	0	2	4	2	4	3
(2) Moderate number of fibroblasts and collagen fibers	1	0	3	2	3	2	3
(3) Small number of fibroblasts and collagen fibers	0	0	1	0	1	0	0
(4) Severe tissue disorganization with necrotic area	0	6	0	0	0	0	0
*Cellular Pattern and Structure of the Bone Tissue*							
(1) Absence of non-vital bone adjacent to the extraction site and trabecular bone filling more than half of the dental alveolus	6	0	0	0	1	0	0

## Data Availability

The original contributions presented in this study are included in the article. Further inquiries can be directed to the corresponding author.

## References

[1] Rogers M. (2003). New Insights Into the Molecular Mechanisms of Action of Bisphosphonates. Curr. Pharm. Des..

[2] Abe K., Yoshimura Y., Deyama Y., Kikuiri T., Hasegawa T., Tei K., Shinoda H., Suzuki K., Kitagawa Y. (2012). Effects of bisphosphonates on osteoclastogenesis in RAW264.7 cells. Int. J. Mol. Med..

[3] Scoletta M., Arduino P.G., Dalmasso P., Broccoletti R., Mozzati M. (2010). Treatment outcomes in patients with bisphosphonate-related osteonecrosis of the jaws: A prospective study. Oral Surg. Oral Med. Oral Pathol. Oral Radiol. Endodontol..

[4] Ebetino F.H., Sun S., Cherian P., Roshandel S., Neighbors J.D., Hu E., Dunford J.E., Sedghizadeh P.P., McKenna C.E., Srinivasan V. (2022). Bisphosphonates: The role of chemistry in understanding their biological actions and structure-activity relationships, and new directions for their therapeutic use. Bone.

[5] Ruggiero S.L., Dodson T.B., Fantasia J., Goodday R., Aghaloo T., Mehrotra B., O’Ryan F. (2014). American Association of Oral and Maxillofacial Surgeons Position Paper on Medication-Related Osteonecrosis of the Jaw—2014 Update. J. Oral Maxillofac. Surg..

[6] Ruggiero S.L., Dodson T.B., Aghaloo T., Carlson E.R., Ward B.B., Kademani D. (2022). American Association of Oral and Maxillofacial Surgeons’ Position Paper on Medication-Related Osteonecrosis of the Jaws—2022 Update. J. Oral Maxillofac. Surg..

[7] Schiodt M., Otto S., Fedele S., Bedogni A., Nicolatou-Galitis O., Guggenberger R., Herlofson B.B., Ristow O., Kofod T. (2019). Workshop of European task force on medication-related osteonecrosis of the jaw—Current challenges. Oral Dis..

[8] Madeira M., Rocha A.C., Moreira C.A., Aguiar Á.M.M., Maeda S.S., Cardoso A.S., Castro C.H.d.M., D’ALva C.B., Silva B.C.C., Ferraz-De-Souza B. (2020). Prevention and treatment of oral adverse effects of antiresorptive medications for osteoporosis—A position paper of the Brazilian Society of Endocrinology and Metabolism (SBEM), Brazilian Society of Stomatology and Oral Pathology (Sobep), and Brazilian Association for Bone Evaluation and Osteometabolism (Abrasso). Arch. Endocrinol. Metab..

[9] Golu M.V., Paşcanu I., Petrovan C., Mocan S., Cosarcă A., Temistocle D.B., Ormenişan A. (2024). Resemblances and differences between osteoradionecrosis of the jaw and medication-related osteonecrosis of the jaw. Med. Pharm. Rep..

[10] Ramalho-Ferreira G., Faverani L.P., Prado F.B., Garcia I.R., Okamoto R. (2015). Raloxifene enhances peri-implant bone healing in osteoporotic rats. Int. J. Oral Maxillofac. Surg..

[11] Ramalho-Ferreira G., Faverani L.P., Momesso G.A.C., Luvizuto E.R., de Oliveira Puttini I., Okamoto R. (2017). Effect of antiresorptive drugs in the alveolar bone healing. A histometric and immunohistochemical study in ovariectomized rats. Clin. Oral Investig..

[12] Faverani L.P., Polo T.O.B., Ramalho-Ferreira G., Momesso G.A.C., Hassumi J.S., Rossi A.C., Freire A.R., Prado F.B., Luvizuto E.R., Gruber R. (2018). Raloxifene but not alendronate can compensate the impaired osseointegration in osteoporotic rats. Clin. Oral Investig..

[13] Ervolino E., Olivo M.B., Toro L.F., de Oliveira Alvarenga Freire J., Ganzaroli V.F., Guiati I.Z., Nuernberg M.A.A., Franciscon J.P.S., Cintra L.T.Â., Garcia V.G. (2022). Effectiveness of antimicrobial photodynamic therapy mediated by butyl toluidine blue in preventing medication-related osteonecrosis of the jaws in rats. Photodiagn. Photodyn. Ther..

[14] Monteiro C.G.J., Vieira E.M., Emerick C., Azevedo R.S., Pascoal V.A.B., Homsi N., Lins R.X. (2021). Ozonated oil effect for prevention of medication-related osteonecrosis of the jaw (MRONJ) in rats undergoing zoledronic acid therapy. Clin. Oral Investig..

[15] Erdemci F., Gunaydin Y., Sencimen M., Bassorgun I., Ozler M., Oter S., Gulses A., Gunal A., Sezgin S., Bayar G. (2014). Histomorphometric evaluation of the effect of systemic and topical ozone on alveolar bone healing following tooth extraction in rats. Int. J. Oral Maxillofac. Surg..

[16] Delanora L.A., de Lima Neto T.J., da Rocha T.E., Silveira G.R.C., Levin L., Shibli J.A., Ervolino E., Mourão C.F., Faverani L.P. (2025). Systemic Ozone Therapy Improves Oral Hard and Soft Tissue Healing in Medication-Related Osteonecrosis of the Jaw (MRONJ): A Study in Senescent Female Rats. Biomedicines.

[17] Ripamonti C.I., Cislaghi E., Mariani L., Maniezzo M. (2011). Efficacy and safety of medical ozone (O3) delivered in oil suspension applications for the treatment of osteonecrosis of the jaw in patients with bone metastases treated with bisphosphonates: Preliminary results of a phase I–II study. Oral Oncol..

[18] Cabras M., Gambino A., Broccoletti R., Sciascia S., Arduino P.G. (2021). Lack of evidence in reducing risk of MRONJ after teeth extractions with systemic antibiotics. J. Oral Sci..

[19] Poi W.R., Carvalho P.S.P., Panzarini S.R., Dezan Júnior E., Trevisan C.L., Souza G. (2000). Efeito da pasta de metronidazol, lidocaína e ascorbosilane C sobre o processo de reparo de alvéolos dentais infectados em ratos. Rev. Bras. Cir. Implant..

[20] Kaur J., Raval R., Bansal A., Kumawat V. (2017). Repercussions of intraalveolar placement of combination of 0.2% chlorhexidine and 10 Mg metronidazole gel on the occurrence of dry sockets—A randomized control trial. J. Clin. Exp. Dent..

[21] Zhou J., Hu B., Liu Y., Yang Z., Song J. (2017). The efficacy of intra-alveolar 0.2% chlorhexidine gel on alveolar osteitis: A meta-analysis. Oral Dis..

[22] Abdullah-AbuMostafa N., Alqahtani A., Abu-Hasna M., Alhokail A., Aladsani A. (2015). A randomized clinical trial compared the effect of intra-alveolar 0.2% Chlorohexidine bio-adhesive gel versus 0.12% Chlorohexidine rinse in reducing alveolar osteitis following molar teeth extractions. Med. Oral Patol. Oral Cir. Bucal.

[23] de Barros Silva P.G., de Lima Praxedes Praxedes Neto R.A., Lima L.A., Lemos J.V.M., Rodrigues M.I.D.Q., Alves A.P.N.N., Dantas T.S., Lima R.A. (2022). Photodynamic therapy and photobiomodulation therapy in zoledronic acid-induced osteonecrosis in rats. Photodiagn. Photodyn. Ther..

[24] Percie du Sert N., Hurst V., Ahluwalia A., Alam S., Avey M.T., Baker M., Browne W.J., Clark A., Cuthill I.C., Dirnagl U. (2020). The ARRIVE guidelines 2.0: Updated guidelines for reporting animal research. PLoS Biol..

[25] Ervolino E., Statkievicz C., Toro L.F., de Mello-Neto J.M., Cavazana T.P., Issa J.P.M., Dornelles R.C.M., de Almeida J.M., Nagata M.J.H., Okamoto R. (2019). Antimicrobial photodynamic therapy improves the alveolar repair process and prevents the occurrence of osteonecrosis of the jaws after tooth extraction in senile rats treated with zoledronate. Bone.

[26] Toro L.F., de Mello-Neto J.M., Santos F.F.V.D., Statkievicz C., Cintra L.T.Â., Issa J.P.M., Dornelles R.C.M., de Almeida J.M., Nagata M.J.H. (2019). Application of Autologous Platelet-Rich Plasma on Tooth Extraction Site Prevents Occurence of Medication-Related Osteonecrosis of the Jaws in Rats. Sci. Rep..

[27] Baggio A.M.P., Bizelli V.F., Delamura I.F., Viotto A.H.A., Veras A.S.C., Teixeira G.R., Faverani L.P., Bassi A.P.F. (2024). Systemic ozone therapy as an adjunctive treatment in guided bone regeneration: A histomorphometrical and immunohistochemical study in rats. Clin. Oral Investig..

[28] Silva M.C., Delamura I.F., de Sá Simon M.E., Barbosa S., Ting D.T., Bechara K., Shibli J.A., Mourão C.F., Bassi A.P.F., Ervolino E. (2024). Is There an Ideal Concentration of Ozonized Oil for the Prevention and Modulation of Zoledronate-Induced Mandibular Osteonecrosis? A Study on Senescent Rats. J. Funct. Biomater..

[29] Miyasawa E.M., Ervolino E., Cardoso J.d.M., Theodoro L.H., Silveira G.R.C., de Molon R.S., Levin L., Garcia V.G., Padovan L.E.M. (2024). Effects of systemic ozone administration on the fresh extraction sockets healing: A histomorphometric and immunohistochemical study in rats. J. Appl. Oral Sci..

[30] Garcia V.G., Longo M., Gualberto Júnior E.C., Bosco A.F., Nagata M.J.H., Ervolino E., Theodoro L.H. (2014). Effect of the Concentration of Phenothiazine Photosensitizers in Antimicrobial Photodynamic Therapy on Bone Loss and the Immune Inflammatory Response of Induced Periodontitis in Rats. J. Periodontal Res..

[31] de Molon R.S., de Avila E.D., Rodrigues J.V.S., Santana A.P., Cunha D.M., Ervolino E., Garcia V.G., Theodoro L.H., Tetradis S. (2025). An evidence-guided review of preclinical strategies to counteract medication-related osteonecrosis of the jaw. What do rodent studies tell us?. JBMR Plus.

[32] Oguz E., Ekinci S., Eroglu M., Bilgic S., Koca K., Durusu M., Kaldirim U., Sadir S., Yurttas Y., Cakmak G. (2011). Evaluation and Comparison of the Effects of Hyperbaric Oxygen and Ozonized Oxygen as Adjuvant Treatments in an Experimental Osteomyelitis Model. J. Surg. Res..

[33] de Medeiros Cardoso J., Ervolino E., Miyasawa E.M., Theodoro L.H., Padovan L.E.M., Pereira E.L., de Molon R.S., Garcia V.G. (2024). Unveiling the Therapeutic Potential of Systemic Ozone on Skin Wound Repair: Clinical, Histological, and Immunohistochemical Study in Rats. BioMed Res. Int..

[34] Lattouf R., Younes R., Lutomski D., Naaman N., Godeau G., Senni K., Changotade S. (2014). Picrosirius Red Staining. J. Histochem. Cytochem..

[35] Alsalih A., Dam A., Lindberg P., Truedsson A. (2021). Medication-Related Osteonecrosis of the Jaws Initiated by Zoledronic Acid and Potential Pathophysiology. Dent. J..

[36] Kuroshima S., Al-Omari F.A., Sasaki M., Sawase T. (2022). Medication-related osteonecrosis of the jaw: A literature review and update. Genesis.

[37] Muthukrishnan A., Bijai Kumar L., Ramalingam G. (2016). Medication-related osteonecrosis of the jaw: A dentist’s nightmare. BMJ Case Rep..

[38] Nicolatou-Galitis O., Kouri M., Papadopoulou E., Vardas E., Galiti D., Epstein J.B., Elad S., Campisi G., Tsoukalas N. (2019). Osteonecrosis of the jaw related to non-antiresorptive medications: A systematic review. Support. Care Cancer.

[39] Kawahara M., Kuroshima S., Sawase T. (2021). Clinical considerations for medication-related osteonecrosis of the jaw: A comprehensive literature review. Int. J. Implant. Dent..

[40] Fleisher K.E., Janal M.N., Albstein N., Young J., Bikhazi V., Schwalb S., Wolff M., Glickman R.S. (2019). Comorbid conditions are a risk for osteonecrosis of the jaw unrelated to antiresorptive therapy. Oral Surg. Oral Med. Oral Pathol. Oral Radiol..

[41] Sculean A., Gruber R., Bosshardt D.D. (2014). Soft tissue wound healing around teeth and dental implants. J. Clin. Periodontol..

[42] Endo Y., Kumamoto H., Nakamura M., Funayama S., Suzuki T., Hattori T., Miyamoto I., Takahashi T., Ueda M., Aoki T. (2017). Mechanisms and Therapeutic Strategies for Bisphosphonate-Related Osteonecrosis of the Jaw (BRONJ). Biol. Pharm. Bull..

[43] da Silva W.P.P., Delanora L.A., Rios B.R., Barbosa S., Simon M.E.S., Sukotjo C., Faverani L.P. (2023). Feasible Low Bone Density Condition for Assessing Bioactivity in Ex-In Vivo and In Vivo Studies. J. Appl. Oral Sci..

[44] El Meligy O.A., Elemam N.M., Talaat I.M. (2023). Ozone therapy in medicine and dentistry: A review of the literature. Dent. J..

[45] Butera A., Pascadopoli M., Gallo S., Martínez C.P.-A., de Val J.E.M.S., Parisi L., Gariboldi A., Scribante A. (2023). Ozonized hydrogels vs. 1% chlorhexidine gel for the clinical and domiciliary management of peri-implant mucositis: A randomized clinical trial. J. Clin. Med..

[46] Jiang A., Zhang Z., Qiu X., Guo Q. (2024). Medication-related osteonecrosis of the jaw (MRONJ): A review of pathogenesis hypothesis and therapy strategies. Arch. Toxicol..

[47] Hernando-Calzado L., Bauer-González A., Cobo-Vázquez C.M., Meniz-García C., López-Quiles J., Madrigal Martínez-Pereda C. (2026). Use of bmps as a treatment for medication-related maxillary osteonecrosis (mronj): A systematic review. Acta Odontol. Scand..

