# Correction: Delamura et al. Parenteral Ozone Therapy Combined with Topical Antimicrobials Enhances Tissue Repair in Mandibular Osteonecrosis of Elderly Female Rats. *J. Funct. Biomater.* 2026, *17*, 378

**DOI:** 10.3390/jfb17090427

**Published:** 2026-08-24

**Authors:** Izabela Fornazari Delamura, Melissa Koto Murai, Arthur Henrique Alécio Viotto, Ana Maira Pereira Baggio, Natália Saori Izumi, Douglas Sadrac de Biagi Ferreira, Vinicius Ferreira Bizelli, Emanuele di Edoardo, Ignazio Scuto, Pietro Montemezzi, Leonardo Perez Faverani, Carlos Fernando Mourão, Ana Paula Farnezi Bassi

**Affiliations:** 1 Department of Diagnosis and Surgery, Aracatuba School of Dentistry, Sao Paulo State University (UNESP), Aracatuba 16015-050, SP, Brazilv.bizelli@unesp.br (V.F.B.); ana.bassi@unesp.br (A.P.F.B.); 2 Department of Biosciences, Piracicaba Dental School, University of Campinas (UNICAMP), Piracicaba 13414-903, SP, Brazil; 3 IRCCS San Raffaele Hospital, Via Olgettina 60, 20132 Milan, Italy; 4 Department of Oral Diagnosis, Division of Oral and Maxillofacial Surgery, Piracicaba Dental School, University of Campinas (UNICAMP), Piracicaba 13414-903, SP, Brazil; 5 Department of Basic and Clinical Translational Sciences, Tufts University School of Dental Medicine, Boston, MA 02111, USA

In the original publication [1], the Institutional Review Board Statement and Informed Consent Statement were not included. These two parts should be added to the backmatter.

**Institutional Review Board Statement:** The animal study protocol was approved by the Animal Ethics Committee (CEUA) of the School of Dentistry of Aracatuba, São Paulo State University (UNESP) (protocol code FOA n^o^ 0268-2022 and date of 30 June 2024).
**Informed Consent Statement:** Not applicable.

The authors state that the scientific conclusions are unaffected. This correction was approved by the Academic Editor. The original publication has also been updated.
